# New Insights into Nutrients for Bone Health and Disease

**DOI:** 10.3390/nu15122648

**Published:** 2023-06-06

**Authors:** Qian Zhang

**Affiliations:** 1 Department of Nutrition and Health, China Agricultural University, Beijing 100193, China; qianzhang@cau.edu.cn; 2 Food Laboratory of Zhongyuan, Luohe 462300, China

Bone health includes the health of bone minerals, mass, geometry, and microstructure. Keeping bone healthy protects from osteoporosis, fragile bones, and bone fractures with aging. In addition to age, other factors that affect bone health are sex, ethnicity, family history, hormone, physical activity, muscle strength, and nutrition [1]. Although bone loss happens more in middle-aged and older women, it can affect young people too. Nutritional disorders and defective dietary components can influence bone health in multiple ways. For example, micronutrients and macronutrients can benefit and hurt bone health depending on exposure, and dietary alcohol is a risk factor for osteoporosis. Although there has been progress in nutritional targets in bone health, many conclusions remain controversial or undiscovered. This Special Issue is focused on uncovering the underlying molecular mechanisms of all kinds of nutritional factors to bone health in patients or animal models. The published paper studied various interesting aspects of nutrition, such as calcium (Ca), vitamin D, vitamin K, and polyphenols, and explored their effect in samples with diversity.

Calcium is the most studied and most crucial nutrition factor in bone homeostasis. According to Mayoclinic [2], the recommended calcium intake for adult men under 70 and adult women under 50 is 1000 mg per day and 1200 mg for older persons. Bone is the biggest organ in the human body containing about 60–70% inorganic mineral, which is mainly composed of hydroxyapatite Ca_10_(PO_4_)_6_(OH)_2_, and 30–40% organic constituents, which is primarily composed of type I collagen [3]. Therefore, calcium is the most abundant mineral element in bone. Hormones such as parathyroid hormone (PTH), fibroblast growth factor 23 (FGF23), calcitonin, sclerostin, and osteocalcin help to maintain the dynamic balance of calcium between bone and serum. Some studies do not support the positive effect of adequate on bone health in aged people, perhaps because of the decreased osteoblast number, weak absorption, or other reasons [4]. Much work needs to be conducted to clarify the calcium intake range with the people’s age, gender, and lifestyle.

It is well known that vitamin D deficiency is a risk factor for osteoporosis. Vitamin D canbe synthesized by the skin after sunshine exposure, and also absorbed via the intestine. Vitamin D is metabolized in the liver to 25[OH]D and then transported to the kidney to develop into its active form, 1,25(OH)_2_D_3_ [calcitriol], which helps with calcium absorption by enhancing the intestinal transcellular calcium transport process [5]. 1,25(OH)_2_D_3_ induces the biosynthesis of the epithelial calcium channel, transient receptor potential vanilloid type 6, and the calcium-binding protein calbindin-D_9k_ [6]. Although it is well recognized that vitamin D helps build bones, the amounts still need investigation. In 2019, a clinical trial published in JAMA found that compared with moderate vitamin D intake, 10 or more times higher vitamin D intake did not result in more bone mineral density or bone strength in healthy adults. The toxicity or harm of the higher dose of vitamin D needs further research [7]. Vitamin K is a cofactor for the gamma-carboxylation of osteocalcin, which attract calcium to promote mineralization and bone formation [8].

Some studies have shown that a particular fruit and vegetable intake volume lowers the risk of bone fracture [9], which might be partially because of their polyphenols. Due to the antioxidative effect, polyphenols protect the oxidative damage caused by the accumulated reactive oxygen species (ROS) in bone marrow, thereby decreasing the risk of osteoporosis. In addition, polyphenols also downregulate inflammatory cytokine, including the osteoclast differentiation factors like RANKL and TNFa, resulting in reduced osteoclast activities and less bone absorption [10]. Polyphenols were divided into four groups: flavonoids, phenolic acids, polyphenolic amides, and others. Each group contains numerous polyphenols and many new polyphenols associated with bone health need to be discovered.

In this Special Issue, research articles highlight novel findings to contribute insight into the dietary nutrients affecting bone health at the molecular and biomedical levels, advancing knowledge in scientific studies and clinical practice.
